# Long-term association of pericardial adipose tissue with incident diabetes and prediabetes: the Coronary Artery Risk Development in Young Adults Study

**DOI:** 10.4178/epih.e2023001

**Published:** 2022-12-03

**Authors:** Minsuk Oh, Wonhee Cho, Dong Hoon Lee, Kara M. Whitaker, Pamela J. Schreiner, James G. Terry, Joon Young Kim

**Affiliations:** 1 Department of Public Health, Baylor University, Waco, TX, USA; 2 Department of Exercise Science, Syracuse University, Syracuse, NY, USA; 3 Department of Nutrition, Harvard T.H. Chan School of Public Health, Boston, MA, USA; 4 Lee Kong Chian School of Medicine, Nanyang Technological University, Singapore, Singapore; 5 Department of Health and Human Physiology, Department of Epidemiology, University of Iowa, Iowa City, IA, USA; 6 Division of Epidemiology and Community Health, University of Minnesota, Twin Cities, MN, USA; 7 Department of Radiology and Radiological Sciences, Vanderbilt University Medical Center, Nashville, TN, USA

**Keywords:** Adipose tissue, Obesity, Prediabetes, Diabetes

## Abstract

**OBJECTIVES:**

We examined whether pericardial adipose tissue (PAT) is predictive of prediabetes and type 2 diabetes over time.

**METHODS:**

In total, 2,570 adults without prediabetes/diabetes from the Coronary Artery Risk Development in Young Adults Study were followed up over 15 years. PAT volume was measured by computed tomography scans, and the new onset of prediabetes/diabetes was examined 5 years, 10 years, and 15 years after the PAT measurements. Multivariable Cox regression models were used to examine the association between the tertile of PAT and incident prediabetes/diabetes up to 15 years later. The predictive ability of PAT (vs. waist circumference [WC], body mass index [BMI], waist-to-height ratio [WHtR]) for prediabetes/diabetes was examined by comparing the area under the receiver operating characteristic curve (AUC).

**RESULTS:**

The highest tertile of PAT was associated with a 1.56 times (95% confidence interval [CI], 1.03 to 2.34) higher rate of diabetes than the lowest tertile; however, no association was found between the highest tertile of PAT and prediabetes in the fully adjusted models, including additional adjustment for BMI or WC. In the fully adjusted models, the AUCs of WC, BMI, WHtR, and PAT for predicting diabetes were not significantly different, whereas the AUC of WC for predicting prediabetes was higher than that of PAT.

**CONCLUSIONS:**

PAT may be a significant predictor of hyperglycemia, but this association might depend on the effect of BMI or WC. Additional work is warranted to examine whether novel adiposity indicators can suggest advanced and optimal information to supplement the established diagnosis for prediabetes/diabetes.

## INTRODUCTION

Obesity is a primary etiology of insulin resistance and diabetes. Specifically, the well-established biological mechanism is that visceral, unlike subcutaneous, adipose tissue secretes free-fatty acids and has higher lipolytic activity, which results in insulin resistance, hyperinsulinemia, and/or diabetes [1-4]. Emerging evidence has also shown that adipose tissue surrounding the heart, which is metabolically akin to and highly correlated with visceral adipose tissue (VAT) [5], may be associated with a higher risk of type 2 diabetes (hereafter “diabetes”) [4,6]. Pericardial adipose tissue (PAT) is an ectopic depot surrounding the coronary arteries, the heart, and the aortic root. PAT secretes inflammatory cytokines into the coronary circulation and more free fatty acids than other adipose tissue depots [5]. As such, PAT is a known risk factor for hyperglycemia, which could potentially have deleterious cardiovascular effects, irrespective of obesity status [5,7].

The association between PAT and diabetes remains inconclusive. A few studies found that the volume of either epicardial adipose tissue (e.g., intra-pericardial adipose tissue) [8] or PAT [4,6] was significantly higher in individuals with prediabetes and diabetes than in normoglycemic individuals, independent of body mass index (BMI), waist circumference (WC), and/or visceral adipose tissue. Conversely, Liu et al. [9] reported that adjustment for VAT attenuated the cross-sectional association between PAT and diabetes. The findings of previous studies are inconsistent in terms of adjustments and limited by cross-sectional study designs. Research with a large sample using a longitudinal study design could clarify the temporality and independence of the associations.

Clinical measures of adiposity, such as WC, BMI, the waist-to-hip ratio, or the waist-to-height ratio (WHtR), have been thoroughly investigated as predictors of diabetes [10-13]. A systematic review and meta-analysis by Kodama et al. [13] concluded that anthropometric indicators of obesity/adiposity (e.g., BMI, WC, the waist-to-hip ratio, WHtR) were statistically significant in predicting diabetes, but WC and WHtR had greater predictive abilities than the other indicators. However, whether PAT predicts long-term incident prediabetes/diabetes and is comparable to the classic anthropometric measures of obesity remains unclear.

The purpose of this study was to examine whether PAT predicts future incident prediabetes/diabetes in individuals without prevalent prediabetes/diabetes. We hypothesized that higher PAT volume is associated with a higher incidence of prediabetes/diabetes over time. Further, we examined variation in the associations by sex, age, race, and/or BMI. Moreover, we aimed to examine the predictive ability of PAT for incident prediabetes/diabetes over 15 years of follow-up in comparison to the predictive abilities of WC, BMI, and WHtR. We hypothesized that the predictive ability of PAT in predicting incident prediabetes/diabetes is comparable to that of WC, BMI, and/or WHtR. Lastly, as an exploratory aim, we examined an optimal cut-off value of PAT for predicting incident prediabetes/diabetes.

## MATERIALS AND METHODS

### Study sample

The Coronary Artery Risk Development in Young Adults (CARDIA) study is a prospective cohort study investigating the trends and correlates of coronary artery disease risk factors and their clinical sequelae. The CARDIA study was initiated in 1985-1986 (year 0), with a total of 5,115 healthy Black and White male and female, aged 18 years to 30 years, who completed an in-person clinical examination and were enrolled from 4 United States metropolitan communities: Birmingham, AL; Minneapolis, MN; Chicago, IL; and Oakland, CA. In the present study, we set CARDIA year 15 (2000-2001) as the baseline because computed tomography (CT) scans were first performed in year 15; therefore, PAT data have been available since 2000-2001. From here on, to avoid any confusion on timeline between ours and other CARDIA papers, we will refer to our baseline as “year 15.” From the 3,671 participants (72% of year 0) who participated in the CARDIA year 15 exam (2000-2001), we additionally excluded participants who were missing data on PAT (n=778), had diabetes or prediabetes (n=125), or were missing data on covariates (n=198) at exam year 15. Thus, a total of 2,570 participants were included in the analysis (Supplementary Material 1).

### Assessment of pericardial adipose tissue

The volume (cm^3^) of PAT at year 15 was quantified on 2.5-mm or 3.0-mm-thick scans of the chest obtained using 64-channel, ECG-gated, multidetector CT scanners (GE Healthcare, Milwaukee, WI at the Birmingham and Oakland centers; Siemens, Erlangen, Germany at the Chicago and Minneapolis centers) [6]. To quantify PAT volume, CT slices 15 mm above and 30 mm below the superior extent of the left main coronary artery were selected using an image processing workstation (OsiriX, Pixmeo, Geneva, Switzerland). The analysts manually segmented the images in this 45-mm block of images and then applied a threshold of -190 to -30 Hounsfield units to isolate adipose tissue. Reader reproducibility was calculated in 156 randomly selected re-reads (intrareader: 49 pairs; inter-reader: 107 pairs). The intra-reader variability was 2.0% and the inter-reader variability was 4.2% [6].

### Assessment of prediabetes/diabetes

Diabetes status was defined based on a combination of (1) self-report of oral hypoglycemic medication or insulin use (at years 0, 7, 10, 15, 20, 25, and 30); (2) measured fasting glucose ≥ 126 mg/dL (at years 0, 7, 10, 15, 20, 25, and 30); (3) a glycated hemoglobin (HbA1c) ≥ 6.5% (at years 20 and 25); or (4) a 2-hour postload glucose ≥ 200 mg/dL during a 75-g oral glucose tolerance test (at years 10, 20, 25, and 30), and prediabetes was defined based on a combination of (1) measured fasting glucose 100-125 mg/dL; (2) a 2-hour postload glucose 140-199 mg/dL; or (3) HbA1c 5.7-6.4% [14,15]. Fasting blood samples drawn by venipuncture were processed at a central laboratory using a standard protocol (Molecular Epidemiology and Biomarker Research Laboratory, University of Minnesota, Minneapolis, MN, USA and Northwest Lipid Research Center, Seattle, WA, USA).

### Other assessments

Sex, age (years), study center, race (Black, White), education (years), occupation status (full time; yes/no), alcohol consumption (mL/day), family history of diabetes (either mother or father; years 0, 5, 10, and 25 only), antihypertensive and blood lipid-lowering medication use, and menopausal status and hormone use (among female only) were assessed using a standard questionnaire. Smoking status (never, former, current) was assessed using a tobacco use questionnaire [16]. BMI was calculated as the measured weight in kilograms divided by height in meters squared. WC was measured to the nearest 0.5 cm in duplicate and averaged using anthropometric tape midway between the iliac crest and the lowest lateral portion of the rib cage. WHtR was calculated as WC divided by height in meters. Systolic and diastolic blood pressure were measured 3 times after 5 minutes of rest (1-minute rest between the last 2 readings) using a Hawksley random-zero sphygmomanometer (Hawksley, Lancing, UK) [17], with the first and fifth phase Korotkoff sounds recorded and the second and third measurements averaged. Diet quality was assessed using the A Priori Diet Quality Score at years 0, 7, and/or 20 only [18]. Diet quality scores were summed across the 10 components for a maximum score of 100, with low values reflecting a poor diet and high values reflecting a healthy diet. If a diet quality score was available from 1 year (i.e., year 0, 7, or 20), the score from the available year was used; otherwise, the average scores from 2 exam or 3 exam years were calculated to account for variation in diet across time and used for analysis [19]. Moderate-to-vigorous intensity physical activity (exercise units) was assessed using the interviewer-administered CARDIA Physical Activity History Questionnaire [20]. Plasma concentrations of total cholesterol and high-density lipoprotein-cholesterol (HDL-C) were determined enzymatically using a Hitachi 917 analyzer (Hitachi, Tokyo, Japan).

### Statistical analysis

Descriptive statistics, including frequency distributions, as well as measures of central tendency and variability, were used to calculate and present the characteristics of participants from years 15 to 30 stratified by tertile of PAT at year 15. The differences between tertiles of PAT were examined using analysis of variance, the Kruskal-Wallis test, or the chi-square test as appropriate.

Person-time (years) was calculated from the number of participants multiplied by 5 years, 10 years, or 15 years because the observation cycle of the outcome variables was 5-year intervals (beginning at year 15) for all participants. The number of participants with prediabetes/diabetes per 1,000 person-years from year 15 by PAT tertile was calculated, and then the incidence rate was calculated as the total number of new cases of prediabetes/diabetes divided by the sum of the person-years. Multivariable-adjusted Cox proportional hazard regression models (PROC PHREG in SAS version 9.4) were used to estimate the hazard ratios (HRs) and 95% confidence intervals (CIs) for incident prediabetes/diabetes 5 years to 15 years later across PAT tertiles and a continuous PAT variable (per 10 cm^3^ increment) at year 15. The covariates were as follows. Model 1 adjusted for sex, race, center, age at year 15, education (years), and occupation status at year 30; and model 2 adjusted for the variables in model 1, plus smoking status, averages (exam years 15, 20, 25, and 30) of physical activity, alcohol consumption (binary; 0 vs. ≥ 0 mL/day), systolic/diastolic blood pressure, total cholesterol, and HDL-C, and diet quality score derived from years 0, 7, or 20, family history of diabetes at year 25, and antihypertensive and lipid-lowering medication use at year 15. Model 3 adjusted for the variables in model 2, plus average values (exam years 15, 20, 25, and 30) of BMI. We adjusted for clinically relevant covariates a priori based on knowledge from previous studies that they may confound the associations between adipose tissue and diabetes. Potential effect modification was examined using multiplicative interactions of sex, age (median split: 32-40, 41-51 years at year 15), race (Black, White), BMI (< 25.0, 25.0-29.9, ≥30.0 kg/m^2^), and WC (<102 vs. 102 cm ≤for male; <88 vs. 88 cm ≤ for female) at year 15 with PAT.

Unadjusted and multivariable-adjusted (model 2 of the Cox regression models) receiver operating characteristic (ROC) curve analysis was used to examine the diagnostic ability of PAT at year 15 for predicting prediabetes/diabetes 5 years to 15 years later by estimating sensitivity, specificity, and area under the ROC curve (AUC). To examine statistical differences in AUC for PAT versus WC, BMI, and WHtR for predicting prediabetes/diabetes, the algorithm developed by DeLong et al. [21] was used. In addition, separate fully-adjusted ROC curve models were analyzed by WC, BMI, and WHtR to examine whether the addition of PAT changed the AUC of each model. For reference, Spearman correlation coefficients between PAT, WC, BMI, and WHtR from year 15 were examined (Supplementary Material 2). To estimate an optimal cut-off value of PAT in predicting prediabetes/diabetes, we examined the balance of sensitivity and specificity with the highest Youden index [22].

In sensitivity analyses, first, we adjusted for WC instead of BMI in the fully adjusted Cox regression models to examine whether PAT predicts incident prediabetes/diabetes independent of a proxy measure of VAT. Second, we examined the association of PAT with hyperglycemia (prediabetes and diabetes). Third, we examined the association of PAT with prediabetes/diabetes defined by fasting glucose only across exam years. Fourth, we examined the association of PAT (continuous variable) with fasting glucose 5 years, 10 years, and 15 years later, separately, using multivariable linear regression. Lastly, we examined the association of PAT using quartile and quintile splits with incident prediabetes/diabetes. All statistical analyses were conducted using SAS version 9.4 (SAS Institute Inc., Cary, NC, USA). A 2-sided p-value < 0.05 was considered statistically significant.

### Ethics statement

All participants provided written informed consent, and the CARDIA study protocols were annually approved by the institutional review board at each CARDIA center (Birmingham, AL; Minneapolis, MN; Chicago, IL; and Oakland, CA; USA).

## RESULTS

The characteristics of the study cohort at year 15 across tertiles of PAT are presented in Table 1. The mean age of the 2,570 eligible participants was 40.3 years, 55.8% were female, and 44.2% were Black. Except for the rate of alcohol consumption and family history of diabetes, all variables were significantly different across the tertiles of PAT (all p<0.05). The correlations of PAT with BMI, WC, and WHtR were 0.59, 0.74, and 0.69, respectively (all p<0.001; Supplementary Material 2). Compared to the participants in the analytic sample, excluded participants were more likely to be Black, younger, and current smokers, reported lower education levels, had higher WC, BMI, WHtR, systolic/diastolic blood pressure, and PAT, and had lower HDL-C levels, diet quality scores, and physical activity scores (all p<0.05; Supplementary Material 3).

The adjusted HRs and 95% CIs of incident prediabetes/diabetes in 5 years to 15 years across tertiles of PAT at year 15 are presented in Table 2. The incidence rates of diabetes and prediabetes were higher in higher tertiles of PAT. In the fully adjusted model, the highest tertile of PAT was 1.56 times (95% CI, 1.03 to 2.34) more likely to have incident diabetes than the lowest tertile of PAT. Each 10-cm^3^ higher PAT volume was associated with a 10.14 times (95% CI, 10.08 to 10.20) higher incidence of diabetes in the fully adjusted model. There were no significant interactions of sex, age, and BMI with the tertile of PAT in the association with incident prediabetes/diabetes. A race interaction in the association with incident prediabetes was found; however, none of the associations were significant in the stratified analyses.

**Figure 1.**
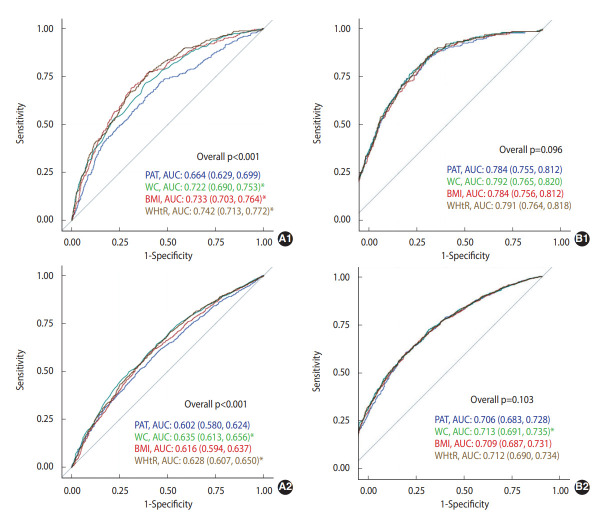
ROC curves of unadjusted (A) and fully adjusted (B) models for PAT versus WC, BMI, WHtR, fasting glucose at exam year 15 in predicting diabetes (1) and prediabetes (2) status 5, 10, and 15 years later, the CARDIA Study (2000-2016). The fully adjusted model adjusted for sex, race, center, age at year 15, education and occupation status at year 30, smoking status at year 30, averages (exam years 15, 20, 25, and 30) of moderate-to-vigorous intensity physical activity, alcohol consumption, systolic blood pressure, diastolic blood pressure, total cholesterol, high-density lipoprotein-cholesterol, diet quality score derived from exam years 0, 7, and/or 20, antihypertensive and lipid-lowering medication use at year 15, and family history of diabetes at year 25. Values are presented as AUC (95% confidence interval). ROC, receiver operating characteristic; WC, waist circumference; WHtR, waist-to-height ratio; AUC, area under the ROC curve; PAT, pericardial adipose tissue; BMI, body mass index; CARDIA, Coronary Artery Risk Development in Young Adults. ^*^p<0.05, statistically significant difference with PAT.

The predictive abilities (AUCs) of PAT, WC, BMI, and WHtR in predicting incident prediabetes/diabetes 5 years to 15 years later are presented in Figure 1. In the unadjusted model predicting diabetes, the AUCs of WC, BMI, and WHtR were significantly higher than that of PAT (p<0.05); however, none were significantly different in the fully adjusted model. The AUCs of WC in predicting incident prediabetes were significantly higher than that of PAT (p<0.05) in both unadjusted and fully adjusted models (the AUC of WHtR was significantly higher than that of PAT).

**Figure 2.**
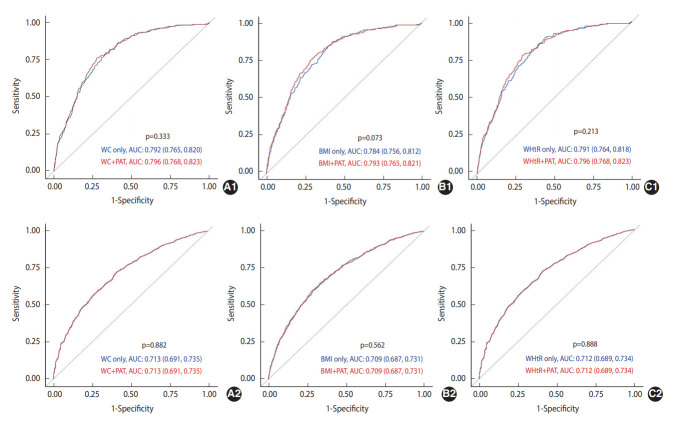
ROC curves of WC (A), BMI (B), and WHtR (C) with additional adjustment for PAT at exam year 15 in predicting diabetes (1) and prediabetes (2) status 5, 10, and 15 years later, the CARDIA Study (2000-2016). All models adjusted for sex, race, center, age at year 15, education and occupation status at year 30, smoking status at year 30, averages (exam years 15, 20, 25, and 30) of moderate-to-vigorous intensity physical activity, alcohol consumption, systolic blood pressure, diastolic blood pressure, total cholesterol, high-density lipoprotein-cholesterol, diet quality score derived from exam years 0, 7, and/or 20, antihypertensive and lipid-lowering medication use at exam year 15, and family history of diabetes at exam year 25. Values are presented as AUC (95% confidence interval). p-values indicate statistically significant differences with PAT. ROC, receiver operating characteristic; WHtR, waist-to-height ratio; AUC, area under the ROC curve; PAT, pericardial adipose tissue; WC, waist circumference; BMI, body mass index; CARDIA, Coronary Artery Risk Development in Young Adults.

The AUCs of WC, BMI, and WHtR, after adjustment for covariates and with and without adjustment for PAT, in predicting incident prediabetes/diabetes at 5 years and 15 years are presented in Figure 2. The AUCs of WC, BMI, and WHtR in predicting incident diabetes improved after adjusting for PAT; however, none were statistically significant. Furthermore, none of the AUCs for predicting incident prediabetes significantly improved after adjusting for PAT.

The highest sensitivity, specificity, and Youden index to estimate the best cut-off values of PAT for predicting prediabetes/diabetes were 34.5 cm^3^/42.3 cm^3^ for female and 51.2 cm^3^/63.1 cm^3^ for male, respectively (Supplementary Material 4). In the fully adjusted model, the association between the highest tertile of PAT with incident diabetes was not significant after adjusting for WC instead of BMI (data not shown). The associations of PAT tertile with incident hyperglycemia (Supplementary Material 5) and fasting glucose-defined prediabetes/diabetes (Supplementary Material 6) were not significant in the fully adjusted models. PAT was significantly associated with high fasting glucose 10 years and 15 years later in the fully adjusted models (Supplementary Material 7). Similar to our findings using tertile splits for classifying PAT, the highest quartile (Supplementary Material 8) and the highest quintile (Supplementary Material 9) of PAT showed significant positive associations with incident diabetes in the fully adjusted models. Lastly, none of the associations of PAT tertiles with incident prediabetes/diabetes were significant for BMI (Supplementary Material 10; except for tertile 2 and incident prediabetes in the BMI category < 25 kg/m^2^) or WC (Supplementary Material 11) stratifications.

## DISCUSSION

In this longitudinal cohort study, high PAT was associated with a higher incidence of diabetes and prediabetes 5 years to 15 years later, independent of the tested covariates; however, these associations were attenuated after additionally adjusting for BMI (for prediabetes) or WC (for diabetes). In addition, the predictive ability of PAT for incident diabetes was comparable to that of WC, BMI, and WHtR when accounting for all the tested covariates. However, the predictive ability of PAT for incident prediabetes was lower than that of WC.

Our findings on the prospective associations of PAT with incident prediabetes/diabetes fill a gap in the current literature that has been limited by cross-sectional observations on prevalent diabetes or higher fasting glucose [6,8,9]. Similar to previous cross-sectional findings, we found that high PAT was associated with a higher incidence of prediabetes/diabetes 5 years to 15 years later. However, after accounting for different adiposity variables in the association of PAT with diabetes, results were mixed across previous studies. For example, Alman et al. [6] found that the highest quartile of PAT was associated with a higher prevalence of diabetes after adjusting for all covariates, including BMI (odds ratio [OR], 2.57; 95% CI, 1.66 to 3.98) or VAT (OR, 2.08; 95% CI, 1.32 to 3.29). Furthermore, Wang et al. [8] found that the highest tertile of epicardial adipose tissue was associated with a higher prevalence of diabetes (OR, 4.82; 95% CI, 1.55 to 16.58) after accounting for all covariates, including WC. On the contrary, Liu et al. [9] found that PAT as a continuous variable was associated with a higher prevalence of diabetes (OR, 1.40; 95% CI, 1.01 to 1.94); however, this association was attenuated after adjusting for BMI or VAT. We found that both the highest tertile of PAT and PAT as a continuous variable were associated with a higher incidence of diabetes after adjusting for BMI (despite a null association when adjusting for WC), and adjusting for BMI attenuated the association between the highest tertile of PAT and prediabetes. Alman et al. [6] suggested that there might be a threshold effect of the highest quartile of PAT in the association with a higher prevalence of diabetes (null findings in quartiles 2 and 3 of PAT). Similarly, our results also appeared to show a threshold effect in the highest tertile of PAT for predicting diabetes (null finding in tertile 2, quartiles 1-3, and quintiles 1-4 of PAT; as shown in Table 3, Supplementary Materials 8 and 9). However, continuous PAT was also associated with both a high risk of incident diabetes and elevated fasting glucose. PAT may be associated with incident diabetes independent of confounders, including BMI; however, WC, as a proxy of visceral adiposity, may confound the association.

Additional longitudinal research is warranted because it is scientifically premature to directly compare our longitudinal results to the previous cross-sectional findings [6,8,9], especially with heterogeneity in measurement matrices (e.g., thickness) and with different approaches to PAT categorization. Bosy-Westphal et al. [23] examined both cross-sectional and longitudinal associations of PAT with insulin resistance and sensitivity in overweight female during approximately 14 weeks of a calorie-restricted diet intervention. PAT was correlated with insulin resistance at baseline (r= 0.46); however, after the 14-week short-term intervention, the longitudinal association between PAT and insulin resistance was not significant.

Although there is no clear consensus as to which adiposity indicators best predict diabetes, WC and WHtR have been recognized as stronger indicators than BMI [10,24,25]. We observed that the AUCs of WC, BMI, and WHtR seemed to be superior to that of PAT for predicting diabetes in crude models. However, interestingly, the AUC of PAT increased and became comparable to those of BMI, WC, and WHtR after adjusting for potential confounders. Furthermore, adding PAT into the BMI and fasting glucose diagnostic models for predicting incident diabetes improved the AUCs (although not to a statistically significant extent). PAT increases progressively with age [26] and is dependent on major health conditions and behaviors [5,27,28]. This suggests that PAT may also be a good adiposity measure, when accounting for socio-demographics and key health behaviors and characteristics, to understand the future risk of diabetes, compared to the wellknown indicators of diabetes [10,24,25]. However, we found that WC was a better indicator than PAT for incident prediabetes, even after adjusting for covariates.

The optimal cut-off values of PAT volume at exam year 15 for predicting incident prediabetes/diabetes 5 years to 15 years later were 34.5 cm^3^/42.3 cm^3^ for female and 51.2 cm^3^/63.1 cm^3^ for male, respectively. A small body of evidence has examined the optimal cut-off values of epicardial adipose tissue thickness in predicting cardiovascular events [29,30] or metabolic syndrome [31,32]. However, to our knowledge, there is no evidence suggesting optimal cut-off values of PAT in predicting prediabetes/diabetes. Additional research with more diverse study populations is needed to confirm our preliminary findings.

The health implications of prediabetes have been underestimated; however, prediabetes is a critical indicator of diabetes risk, and approximately 38% of United States adults ≥ 18 years have prediabetes [33]. As such, early and precise diagnostic actions for identifying prediabetes are important to prevent long-term complications [34]. Overall, we found that the magnitude of associations between PAT and hyperglycemia, and the predictive ability of PAT for hyperglycemia varied by prediabetes/diabetes status. This is possibly because the incidence rate of prediabetes is much higher than that of diabetes, and there may be pathophysiological differences between these conditions [34]. However, there is a lack of evidence about the differences in the predictive ability and the patterns of the association between diagnostic indicators (including PAT) and prediabetes, compared to the associations of those indicators with diabetes.

Strengths of this study include the large sample size with a high participant retention rate, long-term observations of study variables, thorough examinations of potential confounding and effect modifications, a sex/race-balanced cohort, standardized CT measurement of PAT, and repeated screening of glycemic status to define prediabetes/diabetes. However, our study has several limitations. First, the findings cannot be generalized to wider populations given that the study participants were midlife adults residing in the United States. Second, glycemic status was measured every 5 years; therefore, it was not possible to assess the exact timing of the new cases of prediabetes/diabetes. Third, the criteria to define prediabetes/diabetes varied across exam years and 2-hour glucose and HbA1c were not measured at year 15; therefore, it is likely that prediabetes/diabetes might have been underestimated. However, the findings were similar when prediabetes/diabetes was defined with the use of fasting glucose only. Fourth, not only WHtR but also the waist-to-hip ratio may be a good indicator [24,25]. A hip measure was not conducted at years 15 and 20 in CARDIA; therefore, we could not compare the predictive ability of the waist-to-hip ratio. However, WHtR may be superior to the waist-to-hip ratio in predicting prediabetes/diabetes [35]. Lastly, there may have been unmeasured residual confounders that could have interacted with the associations.

In conclusion, a high level of PAT was associated with a higher risk of incident prediabetes/diabetes 5 years to 15 years later, independent of major health characteristics, but this association was attenuated after accounting for BMI or WC. In addition, PAT was comparable to other known anthropometric measures, such as BMI, WC, or WHtR, for predicting incident diabetes. The significance of our findings suggests that PAT can be used as a diagnostic indicator to identify individuals who have prediabetes/diabetes or may be at risk for future diabetes. However, we also suggest that the association of PAT with prediabetes/diabetes may be dependent on BMI or WC. Furthermore, there are health implications of examining the association between PAT and prediabetes/diabetes in that individuals with diabetes are at high risk of cardiovascular events [36] and atherosclerosis [37], resulting from the adverse health consequences of excessive PAT deposition [38].

## Figures and Tables

**Table 1.** Participants characteristics by tertile of PAT at exam year 15, the CARDIA Study (2000-2016)

Characteristics		Total	Tertile of PAT (cm^3^) at exam year 15			
			7.0≤T1≤29.3 (n=856)	29.3<T2≤47.4 (n=857)	47.4<T3 (n=857)	p-value^1^
Female		1,433 (55.8)	621 (72.6)	480 (56.0)	332 (38.7)	<0.001
Age (yr)		40.3±3.6	39.7±3.6	40.2±3.6	40.9±3.5	<0.001
Black		1,136 (44.2)	439 (51.3)	405 (47.3)	525 (61.3)	<0.001
Education (yr)		15.1±2.5	15.4±2.5	15.0±2.4	14.9±2.6	<0.001
Full time occupation		1,924 (74.9)	599 (70.0)	644 (75.2)	681 (79.5)	<0.001
Current smoker						0.016
	Never	1,577 (61.4)	563 (65.8)	516 (60.2)	498 (58.1)	
	Former	487 (19.0)	137 (16.0)	169 (19.7)	181 (21.1)	
	Current	506 (19.7)	156 (18.2)	172 (20.1)	178 (20.8)	
Alcohol consumption (mL/day)		2.4 (13.3)	2.4 (12.1)	2.4 (12.6)	2.4 (16.1)	0.222
	Alcohol consumption	1,368 (53.2)	449 (52.5)	462 (53.9)	457 (53.3)	0.831
Waist circumference (cm)		87.8±13.6	77.1±8.8	87.4±10.6	99.0±11.3	<0.001
Body mass index (kg/m^2^)		28.1±6.1	24.3±4.1	28.0±5.1	31.9±6.4	<0.001
WHtR		0.51±0.1	0.46±0.1	0.51±0.1	0.57±0.1	<0.001
Systolic BP (mmHg)		112.3±14.2	108.9±13.4	112.3±14.3	115.8±14.1	<0.001
Diastolic BP (mmHg)		74.1±11.2	71.5±10.8	74.0±11.0	76.9±11.0	<0.001
Total cholesterol (mg/dL)		184.0±34.0	177.2±31.6	184.0±34.4	191.0±34.5	<0.001
Antihypertensive medication use^2^		161 (6.3)	26 (3.0)	51 (6.0)	84 (9.8)	<0.001
Lipids lowering medication use		48 (1.9)	5 (0.6)	13 (1.5)	30 (3.5)	<0.001
HDL-C (mg/dL)^2^		51.2±14.3	57.3±14.1	51.4±13.7	44.8±12.2	<0.001
Diet quality score^3^		63.1±11.5	64.4±12.3	62.7±11.0	62.2±10.9	<0.001
Self-report MVPA (EU)		288.0 (354.0)	317.5 (382.5)	272.0 (352.0)	276.0 (324.0)	<0.001
PAT (cm^3^) at year 15		43.0±24.2	21.6±5.0	37.7±5.0	69.7±22.6	<0.001
Family history of diabetes^4^		415 (16.2)	136 (15.9)	142 (16.6)	137 (16.0)	0.381

Values are presented as number (%) for categorical variables or mean±standard deviation or median (interquartile range) for continuous variables. PAT, pericardial adipose tissue; CARDIA, Coronary Artery Risk Development in Young Adults; WHtR, waist-to-height ratio; BP, blood pressure; HDL-C, high-density lipoprotein-cholesterol; MVPA, moderate-to-vigorous intensity physical activity; EU, exercise unit.

1 p-value tests for a difference by MVPA tertiles using analysis of variance, Kruskal-Wallis tests, or chi-square tests, as appropriate.

2 n=898.

3 Derived from exam year 0, 7, or 20.

4 n=797; Derived from exam year 25 (either father or mother had diabetes).

**Table 2.** Numbers of incident diabetes/prediabetes 5, 10, and 15 years later by tertile of PAT at exam year 15, the CARDIA Study (2000-2016)

Incident prediabetes/diabetes	Total	Tertile of PAT (cm^3^) at exam year 15			
		7.0≤T1≤29.3 (n=856)	29.3<T2≤47.4 (n=857)	47.4<T3 (n=857)	p-value
Prediabetes at year 20 (n=2,534)	815 (32.2)	205 (24.1)	253 (29.9)	357 (42.7)	<0.001
Diabetes at year 20 (n=2,411)	94 (4.1)	12 (1.6)	29 (3.8)	53 (6.9)	<0.001
Prediabetes at year 25 (n=2,510)	987 (39.3)	287 (33.8)	317 (37.7)	383 (46.7)	<0.001
Diabetes at year 25 (n =2,365)	183 (8.1)	38 (5.1)	43 (5.8)	102 (13.5)	<0.001
Prediabetes at year 30 (n=2,460)	622 (25.3)	148 (17.7)	210 (25.2)	264 (33.5)	<0.001
Diabetes at year 30 (n =2,234)	253 (11.8)	51 (7.0)	65 (9.3)	137 (19.2)	<0.001

Values are presented as number (%). PAT, pericardial adipose tissue; CARDIA, Coronary Artery Risk Development in Young Adults.

**Table 3.** Adjusted hazard ratio (95% confidence interval) of incident diabetes/prediabetes 5, 10, and 15 years later by tertile of PAT at exam year 15, the CARDIA Study (2000-2016)^1^

Values		T1	T2	T3	p_trend_	Per 10 cm^3^ increment
Diabetes 5-15 yr later						
	PYs	12,840	12,855	12,855		
	No. of diabetes	51	65	137		
	Incidence rate^2^	4.0	5.1	10.7		
	Unadjusted	1.00 (reference)	1.28 (0.89, 1.85)	2.82 (2.04, 3.89)^*^	<0.001	10.16 (10.12, 10.20)^*^
	Model 1	1.00 (reference)	1.41 (0.98, 2.05)	3.65 (2.61, 5.12)^*^	<0.001	10.24 (10.20, 10.28)^*^
	Model 2	1.00 (reference)	1.12 (0.77, 1.64)	2.48 (1.72, 3.58)^*^	<0.001	10.19 (10.15, 10.24)^*^
	Model 3	1.00 (reference)	0.91 (0.61, 1.34)	1.56 (1.03, 2.34)^*^	0.004	10.14 (10.08, 10.20)^*^
Prediabetes 5-15 yr later						
	PYs	12,840	12,855	12,855		
	No. of prediabetes	148	210	264		
	Incidence rate^2^	11.5	16.3	20.5		
	Unadjusted	1.00 (reference)	1.45 (1.21, 1.84)^*^	2.07 (1.69, 2.53)	<0.001	10.10 (10.07, 10.13)^*^
	Model 1	1.00 (reference)	1.46 (1.17, 1,80)^*^	1.92 (1.54, 2.38)	<0.001	10.06 (10.06, 10.12)^*^
	Model 2	1.00 (reference)	1.21 (0.97, 1.51)	1.34 (1.06, 1.69)	0.054	10.04 (10.01, 10.08)^*^
	Model 3	1.00 (reference)	1.05 (0.83, 1.31)	0.98 (0.75, 1.27)	0.782	9.99 (9.95, 10.03)

PAT, pericardial adipose tissue; CARDIA, Coronary Artery Risk Development in Young Adults; PY, person-year.

1 PAT (cm^3^) tertile, 7.0≤T1≤29.3 (n=856); 29.3<T2≤47.4 (n=857); 47.4<T3 (n=857); Model 1 adjusts for sex, race, center, age at year 15, education and occupation status at year 30; Model 2 adjusts for model 1, plus smoking status at year 30, averages (exam years 15, 20, 25, and 30) of moderate-to-vigorous intensity physical activity, alcohol, systolic blood pressure, diastolic blood pressure, total cholesterol, high-density lipoprotein-cholesterol, diet quality score (derived from exam years 0, 7, and/or 20), antihypertensive and lipids lowering medication use at year 15, and family history of diabetes at year 25; Model 3 adjusts for model 2, plus body mass index (averages of exam years 15, 20, 25, and 30).

2 Incidence rate indicates per 1,000 PYs.

* p<0.05.

## SUPPLEMENTARY MATERIALS

Supplementary Material 1. Flow diagram of analytical participant selection criteria, the CARDIA Study Abbreviations: CARDIA, coronary artery risk development in young adults; PAT, pericardial adipose tissue.

Supplementary Material 2 Spearman’s (rho) correlation between body mass index, waist circumference, waist-to-height ratio, and pericardial adipose tissue at exam year 15, the CARDIA Study (2000-2001)

Supplementary Material 3 Participants characteristics of those included in analyses at exam year 15 (baseline), compared to those who were excluded, the CARDIA Study (2000-2001)

Supplementary Material 4 Sensitivity, specificity, and Youden index using cut-off values for pericardial adipose tissue at exam years 15 in predicting diabetes and prediabetes 5, 10, and 15 later, the CARDIA Study (2000-2016)

Supplementary Material 5 Adjusted hazard ratio (95% CI) of incident hyperglycemia 5, 10, and 15 years later by tertile of pericardial adipose tissue at exam year 15, the CARDIA Study (2000-2016)

Supplementary Material 6 Adjusted hazard ratio (95% CI) of incident (fasting glucose defined) diabetes/prediabetes 5, 10, and 15 years later by tertile of pericardial adipose tissue at exam year 15, the CARDIA Study (2000-2016)

Supplementary Material 7 Associations of pericardial adipose tissue at year 15 with fasting glucose 5, 10, and 15 years later, the CARDIA Study (2000-2016)

Supplementary Material 8 Adjusted hazard ratio (95% CI) of incident (fasting glucose defined) diabetes/prediabetes 5, 10, and 15 years later by quartile of pericardial adipose tissue at exam year 15, the CARDIA Study (2000-2016)

Supplementary Material 9 Adjusted hazard ratio (95% CI) of incident (fasting glucose defined) diabetes/prediabetes 5, 10, and 15 years later by quintile of pericardial adipose tissue at exam year 15, the CARDIA Study (2000-2016)

Supplementary Material 10 Adjusted hazard ratio (95% CI) of incident diabetes/prediabetes 5, 10, and 15 years later by tertile of pericardial adipose tissue at exam year 15, stratified by body mass index classifications at exam year 15, the CARDIA Study (2000-2016)

Supplementary Material 11 Adjusted hazard ratio (95% CI) of incident diabetes/prediabetes 5, 10, and 15 years later by tertile of pericardial adipose tissue at exam year 15, stratified by waist circumference classifications at exam year 15, the CARDIA Study (2000-2016)
