# Supplementary material for: Long-term association of pericardial adipose tissue with incident diabetes and prediabetes: the Coronary Artery Risk Development in Young Adults Study
Source: Epidemiol Health. 2022 Dec 3;45:e2023001. doi: 10.4178/epih.e2023001 (PMC10106546; doi:10.4178/epih.e2023001)
Supplement: Supplementary Material 1. — Flow diagram of analytical participant selection criteria, the CARDIA Study Abbreviations: CARDIA, coronary artery risk development in young adults; PAT, pericardial adipose tissue. [file epih-45-e2023001-Supplementary-Fig-1.docx]

**Supplementary Material 1.** Flow diagram of analytical participant selection criteria, the CARDIA Study

Abbreviations: CARDIA, coronary artery risk development in young adults; PAT, pericardial adipose tissue.

**
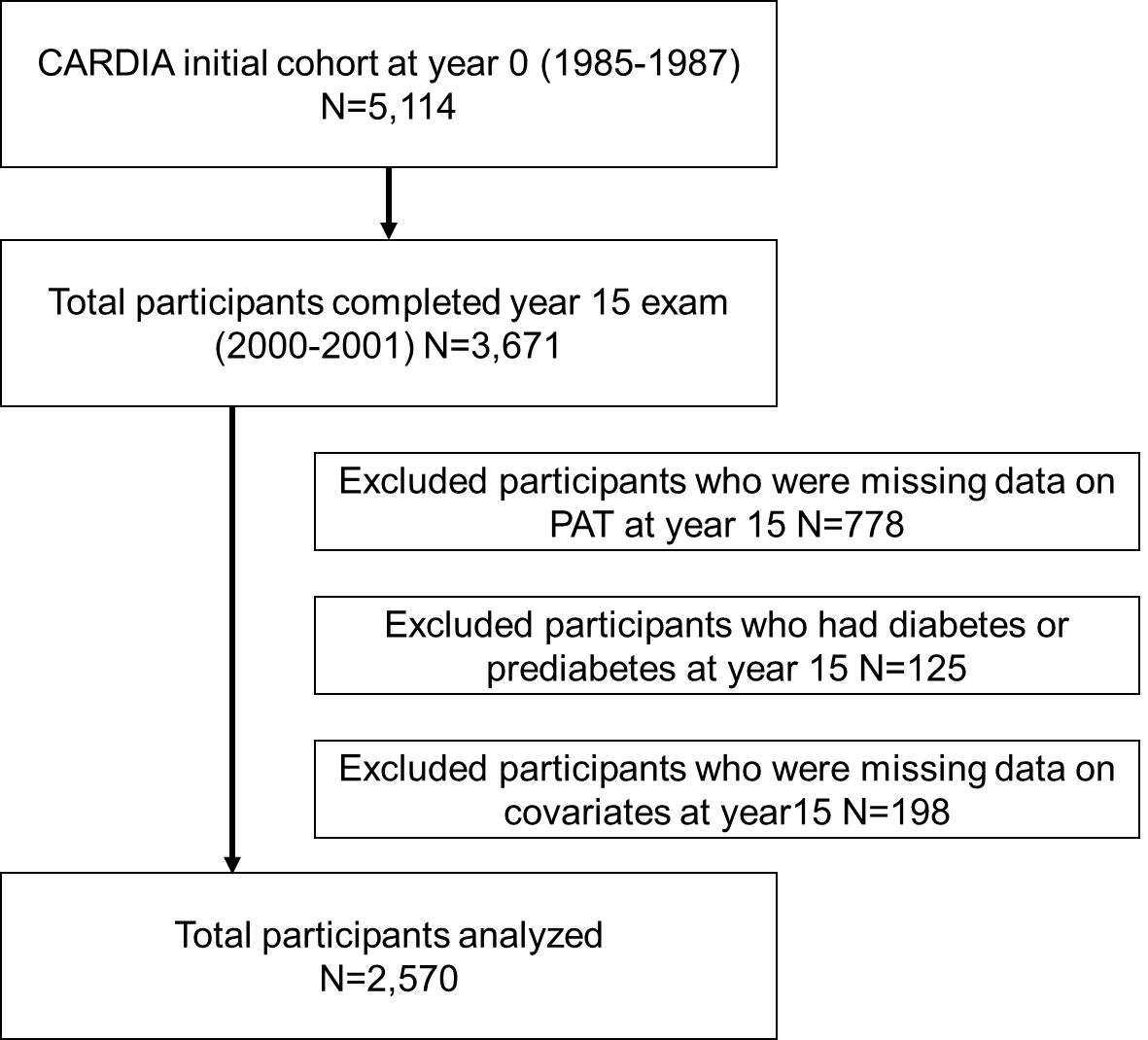
**
