# Supplementary material for: Long-term association of pericardial adipose tissue with incident diabetes and prediabetes: the Coronary Artery Risk Development in Young Adults Study
Source: Epidemiol Health. 2022 Dec 3;45:e2023001. doi: 10.4178/epih.e2023001 (PMC10106546; doi:10.4178/epih.e2023001)
Supplement: Supplementary Material 2 — Spearman’s (rho) correlation between body mass index, waist circumference, waist-to-height ratio, and pericardial adipose tissue at exam year 15, the CARDIA Study (2000-2001) [file epih-45-e2023001-Supplementary-Table-1.docx]

**Supplementary Material 2.** Spearman’s (rho) correlation between body mass index, waist circumference, waist-to-height ratio, and pericardial adipose tissue at exam year 15, the CARDIA Study (2000-2001)

| Variables | BMI | WC | WHtR | PAT |
| --- | --- | --- | --- | --- |
| BMI | - |  |  |  |
| WC | 0.86* | - |  |  |
| WHtR | 0.92* | 0.93* | - |  |
| PAT | 0.59* | 0.74* | 0.69* | - |

*Statistically significant (P < 0.001).

Abbreviations: BMI, body mass index; WC, waist circumference, WHtR, waist-to-height ratio; PAT, pericardial adipose tissue
