# Supplementary material for: Long-term association of pericardial adipose tissue with incident diabetes and prediabetes: the Coronary Artery Risk Development in Young Adults Study
Source: Epidemiol Health. 2022 Dec 3;45:e2023001. doi: 10.4178/epih.e2023001 (PMC10106546; doi:10.4178/epih.e2023001)
Supplement: Supplementary Material 3 — Participants characteristics of those included in analyses at exam year 15 (baseline), compared to those who were excluded, the CARDIA Study (2000-2001) [file epih-45-e2023001-Supplementary-Table-2.docx]

**Supplementary Material 3**. Participants characteristics of those included in analyses at exam year 15 (baseline), compared to those who were excluded, the CARDIA Study (2000-2001)

| Participants Characteristics | n | Included  participants | n | Excluded participants |
| --- | --- | --- | --- | --- |
| Women, n (%) | 2,570 | 1,433 (55.8) | 2,544 | 1,354 (53.2) |
| Black, n (%) | 2,570 | 1,136 (44.2) | 2,544 | 1,501 (59.0)* |
| Age, years | 2,570 | 40.3±3.6 | 1,101 | 40.0±3.7* |
| Education, years | 2,570 | 15.1±2.5 | 1,090 | 14.4±2.5* |
| Full time occupation, n (%) | 2,570 | 1,924 (74.9) | 1,091 | 791 (72.5) |
| Current smoker, n (%) | 2,570 |  | 1,094 | * |
| Never |  | 1,577 (61.4) |  | 615 (56.2) |
| Former |  | 487 (19.0) |  | 178 (16.3) |
| Current |  | 506 (19.7) |  | 301 (27.5) |
| Alcohol consumption, mL/day | 2,570 | 2.4 (13.3) | 1,091 | 0.0 (13.3) |
| Waist circumference, cm | 2,570 | 87.8±13.6 | 1,074 | 92.6±18.9* |
| Body mass index, kg/m^2^ | 2,570 | 28.1±6.1 | 1,058 | 30.4±8.1* |
| WHtR | 2,570 | 0.51±0.1 | 1,060 | 0.55±0.1* |
| Systolic BP, mm Hg | 2,570 | 112.3±14.2 | 1,087 | 115.4±16.3* |
| Diastolic BP, mm Hg | 2,570 | 74.1±11.2 | 1,087 | 75.4±12.5* |
| Total cholesterol, mg/dL | 2,570 | 184.0±34.0 | 1,042 | 186.2±39.8 |
| HDL-C, mg/dL | 2,570 | 51.2±14.3 | 1,042 | 49.5±15.1* |
| ^a^Diet quality score | 2,570 | 63.1±11.5 | 2,493 | 60.8±11.6* |
| Self-reported MVPA, EU | 2,570 | 288.0 (354.0) | 1,084 | 263.5 (343.5)* |
| PAT, mL | 2,570 | 37.6 (28.0) | 323 | 52.2 (43.7)* |

Note: Data are mean ± standard deviation or median (interquartile range) at year 15 unless otherwise specified. ^a^Derived from year 0, 7, or 20. *P < 0.05; p-values testing for differences between Included vs. Excluded using independent samples t-tests, Wilcoxon-Mann Whitney tests, or chi-square tests, as appropriate.

Abbreviations: WHtR, waist-to-height ratio; BP, blood pressure; HDL-C, high-density lipoprotein-cholesterol; MVPA, moderate-to-vigorous intensity physical activity; EU, exercise unit; PAT, pericardial adipose tissue.
