# Supplementary material for: Long-term association of pericardial adipose tissue with incident diabetes and prediabetes: the Coronary Artery Risk Development in Young Adults Study
Source: Epidemiol Health. 2022 Dec 3;45:e2023001. doi: 10.4178/epih.e2023001 (PMC10106546; doi:10.4178/epih.e2023001)
Supplement: Supplementary Material 4 — Sensitivity, specificity, and Youden index using cut-off values for pericardial adipose tissue at exam years 15 in predicting diabetes and prediabetes 5, 10, and 15 later, the CARDIA Study (2000-2016) [file epih-45-e2023001-Supplementary-Table-3.docx]

**Supplementary Material 4**. Sensitivity, specificity, and Youden index using cut-off values for pericardial adipose tissue at exam years 15 in predicting diabetes and prediabetes 5, 10, and 15 later, the CARDIA Study (2000-2016)

| Female | Outcome | Cut-off (cm^3^) | Sensitivity (%) | Specificity (%) | Youden index |
| --- | --- | --- | --- | --- | --- |
|  | Diabetes | **42.3** | 58.8 | 73.9 | 0.33 |
|  | Prediabetes | **34.5** | 51.9 | 63.5 | 0.15 |
| Male | Outcome | Cut-off (cm^3^) | Sensitivity (%) | Specificity (%) | Youden index |
|  | Diabetes | **63.1** | 49.1 | 76.8 | 0.26 |
|  | Prediabetes | **51.2** | 46.7 | 66.6 | 0.14 |

Abbreviations: PAT, pericardial adipose tissue.
