# Supplementary material for: Long-term association of pericardial adipose tissue with incident diabetes and prediabetes: the Coronary Artery Risk Development in Young Adults Study
Source: Epidemiol Health. 2022 Dec 3;45:e2023001. doi: 10.4178/epih.e2023001 (PMC10106546; doi:10.4178/epih.e2023001)
Supplement: Supplementary Material 5 — Adjusted hazard ratio (95% CI) of incident hyperglycemia 5, 10, and 15 years later by tertile of pericardial adipose tissue at exam year 15, the CARDIA Study (2000-2016) [file epih-45-e2023001-Supplementary-Table-4.docx]

**Supplementary Material 5.** Adjusted hazard ratio (95% CI) of incident hyperglycemia 5, 10, and 15 years later by tertile of pericardial adipose tissue at exam year 15, the CARDIA Study (2000-2016)

|  | Hyperglycemia 5 - 15 years later | | | | |
| --- | --- | --- | --- | --- | --- |
|  | T1 | T2 | T3 | P_trend_ | Per 10 cm^3^ increment |
| Person-years | 12,840 | 12,855 | 12,855 |  |  |
| No. of hyperglycemia | 174 | 249 | 351 |  |  |
| Incidence rate* | 13.6 | 19.4 | 27.3 |  |  |
| Unadjusted | 1 (ref.) | **1.50 (1.23, 1.82)** | **2.36 (1.97, 2.84)** | <0.001 | **10.10 (10.10, 10.15)** |
| Model 1 | 1 (ref.) | **1.49 (1.22, 1.82)** | **2.31 (1.90, 2.81)** | <0.001 | **10.13 (10.01, 13.56)** |
| Model 2 | 1 (ref.) | 1.21 (0.98, 1.48) | **1.59 (1.29, 1.97)** | <0.001 | **10.08 (10.05, 10.11)** |
| Model 3 | 1 (ref.) | 1.03 (0.84, 1.27) | 1.13 (0.89, 1.44) | 0.473 | 10.03 (9.99, 10.06) |

Note: Pericardial adipose tissue (cm^3^) tertile: 7.0 ≤T1≤ 29.3, 29.3<T2 ≤47.4, and 47.4<T3. Bolded are statistically significant (P < 0.05). Model 1 adjusts for sex, race, center, age at year 15, education and occupation status at year 20 or 25. Model 2 adjusts for Model 1, plus smoking status at year 20 or 25, averages (exam years 15 and 20, or exam years 15, 20, and 25) of moderate-to-vigorous intensity physical activity, alcohol, systolic blood pressure, diastolic blood pressure, total cholesterol, high-density lipoprotein-cholesterol, antihypertensive and lipids lowering medication use at exam year 15, diet quality score (derived from exam years 0, 7, and/or 20), and family history of diabetes at exam year 25. Model 3 adjusts for Model 2, plus body mass index (averages of exam years 15 and 20, or exam years 15, 20, and 25). *Incidence rate indicates per 1,000 person-years.
