# Supplementary material for: Long-term association of pericardial adipose tissue with incident diabetes and prediabetes: the Coronary Artery Risk Development in Young Adults Study
Source: Epidemiol Health. 2022 Dec 3;45:e2023001. doi: 10.4178/epih.e2023001 (PMC10106546; doi:10.4178/epih.e2023001)
Supplement: Supplementary Material 6 — Adjusted hazard ratio (95% CI) of incident (fasting glucose defined) diabetes/prediabetes 5, 10, and 15 years later by tertile of pericardial adipose tissue at exam year 15, the CARDIA Study (2000-2016) [file epih-45-e2023001-Supplementary-Table-5.docx]

**Supplementary Material 6.** Adjusted hazard ratio (95% CI) of incident (fasting glucose defined) diabetes/prediabetes 5, 10, and 15 years later by tertile of pericardial adipose tissue at exam year 15, the CARDIA Study (2000-2016)

|  | Diabetes 5 - 15 years later^3^ | | | | | | | | | |  |
| --- | --- | --- | --- | --- | --- | --- | --- | --- | --- | --- | --- |
|  | T1 | | | T2 | T3 | | P_trend_ | | Per 10 cm^3^ increment | |  |
| Person-years | 12,990 | | | 12,990 | 12,990 | |  | |  | |  |
| No. of diabetes | 20 | | | 23 | 76 | |  | |  | |  |
| Incidence rate* | 1.5 | | | 1.8 | 5.9 | |  | |  | |  |
| Unadjusted | 1 (ref.) | | | 1.17 (0.64, 2.12) | **3.95 (2.42, 6.47)** | | <0.001 | | **10.19 (10.14, 10.24)** | |  |
| Model 1 | 1 (ref.) | | | 1.20 (0.66, 2.19) | **4.44 (2.65, 7.44)** | | <0.001 | | **10.23 (10.18, 10.29)** | |  |
| Model 2 | 1 (ref.) | | | 0.86 (0.46, 1.63) | **2.60 (1.48, 4.55)** | | <0.001 | | **10.18 (10.12, 10.25)** | |  |
| Model 3 | 1 (ref.) | | | 0.69 (0.36, 1.32) | 1.61 (0.87, 2.99) | | 0.006 | | 10.12 (10.05, 10.20) | |  |
|  | | Prediabetes 5 - 15 years later^3^ | | | | | | | | | |
|  | | T1 | T2 | | | T3 | | P_trend_ | | Per 10 cm^3^ increment | |
| Person-years | | 12,990 | 12,990 | | | 12,990 | |  | |  | |
| No. of prediabetes | | 132 | 182 | | | 256 | |  | |  | |
| Incidence rate* | | 10.2 | 14.0 | | | 19.7 | |  | |  | |
| Unadjusted | | 1 (ref.) | **1.44 (1.15, 1.80)** | | | **2.30 (1.86, 2.84)** | | <0.001 | | **10.11 (10.09, 10.14)** | |
| Model 1 | | 1 (ref.) | **1.34 (1.07, 1.69)** | | | **1.98 (1.58, 2.48)** | | <0.001 | | **10.09 (10.06, 10.12)** | |
| Model 2 | | 1 (ref.) | 1.12 (0.89, 1.42) | | | **1.36 (1.06, 1.75)** | | 0.035 | | **10.04 (10.01, 10.08)** | |
| Model 3 | | 1 (ref.) | 0.97 (0.76, 1.24) | | | 0.98 (0.74, 1.30) | | 0.965 | | 9.98 (9.94, 10.03) | |

Note: Pericardial adipose tissue (cm^3^) tertile: 7.0 ≤T1≤ 29.3, 29.3<T2 ≤47.4, and 47.4<T3. Bolded values are statistically significant (P < 0.05). Model 1 adjusts for sex, race, center, age at exam year 15, education and occupation status at exam year 30. Model 2 adjusts for Model 1, plus smoking status at exam year 30, averages (exam years 15, 20, 25, and 30) of moderate-to-vigorous intensity physical activity, alcohol, systolic blood pressure, diastolic blood pressure, total cholesterol, high-density lipoprotein-cholesterol, diet quality score (derived from exam years 0, 7, and/or 20), antihypertensive and lipids lowering medication use at exam year 15, and family history of diabetes at exam year 25. Model 3 adjusts for Model 2, plus body mass index (averages of exam years 15, 20, 25, and 30). *Incidence rate indicates per 1,000 person-years.
