# Supplementary material for: Long-term association of pericardial adipose tissue with incident diabetes and prediabetes: the Coronary Artery Risk Development in Young Adults Study
Source: Epidemiol Health. 2022 Dec 3;45:e2023001. doi: 10.4178/epih.e2023001 (PMC10106546; doi:10.4178/epih.e2023001)
Supplement: Supplementary Material 7 — Associations of pericardial adipose tissue at year 15 with fasting glucose 5, 10, and 15 years later, the CARDIA Study (2000-2016) [file epih-45-e2023001-Supplementary-Table-6.docx]

**Supplementary Material 7.** Associations of pericardial adipose tissue at year 15 with fasting glucose 5, 10, and 15 years later**,** the CARDIA Study (2000-2016)

|  | Outcome variable: fasting glucose (mg/dL) | | | | | |
| --- | --- | --- | --- | --- | --- | --- |
|  | Exam year 20 | | Exam year 25 | | Exam year 30 | |
|  | β (SE) | 95% CI | β (SE) | 95% CI | β (SE) | 95% CI |
| Unadjusted | **0.13 (0.01)** | 0.11, 0.15 | **0.18 (0.01)** | 0.15, 0.21 | **0.23 (0.02)** | 0.19, 0.27 |
| Model 1 | **0.12 (0.01)** | 0.09, 0.14 | **0.17 (0.02)** | 0.14, 0.20 | **0.23 (0.02)** | 0.18, 0.27 |
| Model 2 | **0.08 (0.01)** | 0.05, 0.10 | **0.11 (0.02)** | 0.08, 0.15 | **0.18 (0.02)** | 0.13, 0.22 |
| Model 3 | 0.03 (0.02) | 0.00, 0.06 | **0.06 (0.02)** | 0.02, 0.10 | **0.11 (0.03)** | 0.06, 0.16 |

Note: Bolded values are statistically significant (P < 0.05). Model 1 adjusts for sex, race, center, age at year 15, education and occupation status at exam years 20 (for 5 years later model), 25 (for 10 years later model), or 30 (for 15 years later model). Model 2 adjusts for Model 1, plus smoking status at exam years 20 (for 5 years later model), 25 (for 10 years later model), or 30 (for 15 years later model), averages (between exam year 15 and exam year 20 (for 5 years later model), 25 (for 10 years later model), or 30 (for 15 years later model)) of moderate-to-vigorous intensity physical activity, alcohol, systolic blood pressure, diastolic blood pressure, total cholesterol, high-density lipoprotein-cholesterol, diet quality score (derived from exam years 0, 7, and/or 20), antihypertensive and lipids lowering medication use at exam year 15, and family history of diabetes at exam year 25 (for 10 years and 15 years later models). Model 3 adjusts for Model 2, plus average of body mass index (between exam year 15 and exam year 20 (for 5 years later model), 25 (for 10 years later model), or 30 (for 15 years later model).
