# Supplementary material for: Long-term association of pericardial adipose tissue with incident diabetes and prediabetes: the Coronary Artery Risk Development in Young Adults Study
Source: Epidemiol Health. 2022 Dec 3;45:e2023001. doi: 10.4178/epih.e2023001 (PMC10106546; doi:10.4178/epih.e2023001)
Supplement: Supplementary Material 8 — Adjusted hazard ratio (95% CI) of incident (fasting glucose defined) diabetes/prediabetes 5, 10, and 15 years later by quartile of pericardial adipose tissue at exam year 15, the CARDIA Study (2000-2016) [file epih-45-e2023001-Supplementary-Table-7.docx]

**Supplementary Material 8.** Adjusted hazard ratio (95% CI) of incident (fasting glucose defined) diabetes/prediabetes 5, 10, and 15 years later by quartile of pericardial adipose tissue at exam year 15, the CARDIA Study (2000-2016)

|  | Diabetes 5 - 15 years later | | | | | | | | | | |
| --- | --- | --- | --- | --- | --- | --- | --- | --- | --- | --- | --- |
|  | Q1 | | | Q2 | Q3 | | Q4 | | P_trend_ | | |
| Person-years | 9,630 | | | 9,645 | 9,645 | | 9,630 | |  | | |
| No. of diabetes | 35 | | | 36 | 68 | | 114 | |  | | |
| Incidence rate* | 3.6 | | | 3.7 | 7.1 | | 11.8 | |  | | |
| Unadjusted | 1 (ref.) | | | 1.03 (0.65, 1.64) | **1.98 (1.31, 2.97)** | | **3.52 (2.41, 5.14)** | | <0.001 | | |
| Model 1 | 1 (ref.) | | | 1.13 (0.71, 1.81) | **2.24 (1.48, 3.38)** | | **5.19 (3.49, 7.72)** | | <0.001 | | |
| Model 2 | 1 (ref.) | | | 0.91 (0.57, 1.48) | **1.63 (1.06, 2.52)** | | **3.45 (2.25, 5.29)** | | <0.001 | | |
| Model 3 | 1 (ref.) | | | 0.82 (0.51, 1.33) | 1.22 (0.77, 1.92) | | **2.20 (1.36, 3.57)** | | <0.001 | | |
|  | | Prediabetes 5 - 15 years later | | | | | | | | | |
|  | | Q1 | Q2 | | | Q3 | | Q4 | | P_trend_ |  |
| Person-years | | 9,630 | 9,645 | | | 9,645 | | 9,630 | |  |  |
| No. of prediabetes | | 104 | 147 | | | 174 | | 197 | |  |  |
| Incidence rate* | | 10.8 | 15.2 | | | 18.0 | | 20.5 | |  |  |
| Unadjusted | | 1 (ref.) | **1.44 (1.12, 1.85)** | | | **1.71 (1.33, 2.19)** | | **2.09 (1.62, 2.70)** | | <0.001 |  |
| Model 1 | | 1 (ref.) | **1.45 (1.12, 1.87)** | | | **1.34 (1.07, 1.69)** | | **1.98 (1.58, 2.48)** | | <0.001 |  |
| Model 2 | | 1 (ref.) | 1.22 (0.94, 1.58) | | | 1.27 (0.98, 1.65) | | **1.38 (1.05, 1.82)** | | 0.150 |  |
| Model 3 | | 1 (ref.) | 1.09 (0.84, 1.41) | | | 0.99 (0.75, 1.31) | | 0.93 (0.68, 1.27) | | 0.680 |  |


Note: Pericardial adipose tissue (cm^3^) quartile: 7.0 ≤ Q1 (n=642) ≤ 25.9, 25.9 < Q2 (n=643) ≤ 37.6, 37.6 < Q3 (n=643) ≤ 53.9, and 53.9 < Q4 (n=642). Bolded values are statistically significant (P < 0.05). Model 1 adjusts for sex, race, center, age at exam year 15, education and occupation status at exam year 30. Model 2 adjusts for Model 1, plus smoking status at exam year 30, averages (exam years 15, 20, 25, and 30) of moderate-to-vigorous intensity physical activity, alcohol, systolic blood pressure, diastolic blood pressure, total cholesterol, high-density lipoprotein-cholesterol, diet quality score (derived from exam years 0, 7, and/or 20), antihypertensive and lipids lowering medication use at exam year 15, and family history of diabetes at exam year 25. Model 3 adjusts for Model 2, plus body mass index (averages of exam years 15, 20, 25, and 30). *Incidence rate indicates per 1,000 person-years.
