# Supplementary material for: Long-term association of pericardial adipose tissue with incident diabetes and prediabetes: the Coronary Artery Risk Development in Young Adults Study
Source: Epidemiol Health. 2022 Dec 3;45:e2023001. doi: 10.4178/epih.e2023001 (PMC10106546; doi:10.4178/epih.e2023001)
Supplement: Supplementary Material 9 — Adjusted hazard ratio (95% CI) of incident (fasting glucose defined) diabetes/prediabetes 5, 10, and 15 years later by quintile of pericardial adipose tissue at exam year 15, the CARDIA Study (2000-2016) [file epih-45-e2023001-Supplementary-Table-8.docx]

**Supplementary Material 9.** Adjusted hazard ratio (95% CI) of incident (fasting glucose defined) diabetes/prediabetes 5, 10, and 15 years later by quintile of pericardial adipose tissue at exam year 15, the CARDIA Study (2000-2016)

|  | Diabetes 5 - 15 years later | | | | | | | | | |
| --- | --- | --- | --- | --- | --- | --- | --- | --- | --- | --- |
|  | Q1 | | | Q2 | Q3 | Q4 | | | Q5 | P_trend_ |
| Person-years | 7,710 | | | 7,710 | 7,710 | 7,710 | | | 7,710 |  |
| No. of diabetes | 28 | | | 32 | 39 | 53 | | | 101 |  |
| Incidence rate* | 3.6 | | | 4.2 | 5.1 | 6.9 | | | 13.1 |  |
| Unadjusted | 1 (ref.) | | | 1.15 (0.86, 2.28) | 1.40 (0.86, 2.28) | **1.94 (1.22, 3.06)** | | | **3.95 (2.60, 6.00)** | <0.001 |
| Model 1 | 1 (ref.) | | | 1.23 (0.74, 2.04) | 1.58 (0.97, 2.58) | **2.31 (1.45, 3.68)** | | | **6.03 (3.89, 9.33)** | <0.001 |
| Model 2 | 1 (ref.) | | | 1.06 (0.63, 1.77) | 1.23 (0.74, 2.04) | **1.68 (1.04, 2.73)** | | | **3.93 (2.46, 6.30)** | <0.001 |
| Model 3 | 1 (ref.) | | | 0.94 (0.56, 1.59) | 0.98 (0.59, 1.65) | 1.17 (0.70, 1.20) | | | **2.41 (1.42, 4.08)** | <0.001 |
|  | | Prediabetes 5 - 15 years later | | | | | | | | |
|  | | Q1 | Q2 | | Q3 | | Q4 | Q5 | | P_trend_ |
| Person-years | | 7,710 | 7,710 | | 7,710 | | 7,710 | 7,710 | |  |
| No. of prediabetes | | 82 | 103 | | 126 | | 150 | 161 | |  |
| Incidence rate* | | 10.6 | 13.4 | | 16.3 | | 19.5 | 20.9 | |  |
| Unadjusted | | 1 (ref.) | 1.31 (0.98, 1.75) | | **1.62 (1.23, 2.14)** | | **2.03 (1.55, 2.66)** | **2.40 (1.84, 3.13)** | | <0.001 |
| Model 1 | | 1 (ref.) | 1.32 (0.99, 1.77) | | **1.56 (1.18, 2.07)** | | **1.91 (1.45, 2.51)** | **2.24 (1.69, 2.97)** | | <0.001 |
| Model 2 | | 1 (ref.) | 1.16 (0.87, 1.56) | | 1.22 (0.92, 1.63) | | **1.37 (1.03, 1.82)** | **1.42 (1.05, 1.93)** | | 0.181 |
| Model 3 | | 1 (ref.) | 1.05 (0.78, 1.41) | | 1.02 (0.76, 1.37) | | 1.02 (0.75, 1.39) | 0.93 (0.66, 1.32) | | 0.936 |


Note: Pericardial adipose tissue (cm^3^) quartile: 7.0 ≤ Q1 (n=514) ≤ 23.8, 23.8 < Q2 (n=514) ≤ 32.7, 32.7 < Q3 (n=514) ≤ 43.2, 43.2 < Q4 (n=514) ≤ 58.8, and 58.8 < Q5 (n=514). Bolded values are statistically significant (P < 0.05). Model 1 adjusts for sex, race, center, age at exam year 15, education and occupation status at exam year 30. Model 2 adjusts for Model 1, plus smoking status at exam year 30, averages (exam years 15, 20, 25, and 30) of moderate-to-vigorous intensity physical activity, alcohol, systolic blood pressure, diastolic blood pressure, total cholesterol, high-density lipoprotein-cholesterol, diet quality score (derived from exam years 0, 7, and/or 20), antihypertensive and lipids lowering medication use at exam year 15, and family history of diabetes at exam year 25. Model 3 adjusts for Model 2, plus body mass index (averages of exam years 15, 20, 25, and 30). *Incidence rate indicates per 1,000 person-years.
