# Supplementary material for: Long-term association of pericardial adipose tissue with incident diabetes and prediabetes: the Coronary Artery Risk Development in Young Adults Study
Source: Epidemiol Health. 2022 Dec 3;45:e2023001. doi: 10.4178/epih.e2023001 (PMC10106546; doi:10.4178/epih.e2023001)
Supplement: Supplementary Material 11 — Adjusted hazard ratio (95% CI) of incident diabetes/prediabetes 5, 10, and 15 years later by tertile of pericardial adipose tissue at exam year 15, stratified by waist circumference classifications at exam year 15, the CARDIA Study (2000-2016) [file epih-45-e2023001-Supplementary-Table-10.docx]

**Supplementary Material 11.** Adjusted hazard ratio (95% CI) of incident diabetes/prediabetes 5, 10, and 15 years later by tertile of pericardial adipose tissue at exam year 15, stratified by waist circumference classifications at exam year 15, the CARDIA Study (2000-2016)

| Men | | | | |
| --- | --- | --- | --- | --- |
| WC classification | Diabetes 5 - 15 years later | | | |
|  | T1 | T2 | T3 | P_trend_ |
| WC < 102 cm | 1 (ref.) | 1.03 (0.47, 2.27) | 1.66 (0.74, 3.69) | 0.289 |
| 102 cm ≤ WC | 1 (ref.) | 0.65 (0.32, 1.58) | 1.21 (0.59, 2.64) | 0.033 |
| WC classification | Prediabetes 5 - 15 years later | | | |
|  | T1 | T2 | T3 | P_trend_ |
| WC < 102 cm | 1 (ref.) | 0.96 (0.66, 1.41) | 0.99 (0.67, 1.47) | 0.968 |
| 102 cm ≤ WC | 1 (ref.) | 1.31 (0.16, 10.66) | 0.56 (0.07, 4.61) | 0.070 |
| Women | | | | |
| WC classification | Diabetes 5 - 15 years later | | | |
|  | T1 | T2 | T3 | P_trend_ |
| WC < 88 cm | 1 (ref.) | 1.24 (0.67, 2.30) | 1.96 (0.73, 5.30) | 0.397 |
| 88 cm ≤ WC | 1 (ref.) | 0.76 (0.36, 1.60) | 1.41 (0.70, 2.83) | 0.334 |
| WC classification | Prediabetes 5 - 15 years later | | | |
|  | T1 | T2 | T3 | P_trend_ |
| WC < 88 cm | 1 (ref.) | 1.30 (0.92, 1.82) | 1.10 (0.60, 2.02) | 0.322 |
| 88 cm ≤ WC | 1 (ref.) | 1.08 (0.55, 2.12) | 1.34 (0.70, 2.56) | 0.379 |

Note: Pericardial adipose tissue (cm^3^) tertile, 7.0≤T1≤29.3 (n=856); 29.3<T2≤47.4 (n=857); 47.4<T3 (n=857). WC, waist circumference. Bolded values are statistically significant (P < 0.05). Models adjust for race, center, age at year 15, education and occupation status at year 30, smoking status at year 30, averages (exam years 15, 20, 25, and 30) of moderate-to-vigorous intensity physical activity, alcohol, systolic blood pressure, diastolic blood pressure, total cholesterol, high-density lipoprotein-cholesterol, diet quality score (derived from exam years 0, 7, and/or 20), antihypertensive and lipids lowering medication use at year 15, and family history of diabetes at year 25.
